# LncRNA CHRF: molecular mechanisms and therapeutic potentials in cardiovascular diseases, cancers and fibrosis

**DOI:** 10.3389/fcell.2025.1573723

**Published:** 2025-06-19

**Authors:** Jie Mou, Chuntao Luo, Wen Zhang, Yin Shao, Juan Pei, Yaqin Chen, Xin Guo, Yonghong Fan, Hongyu Sun

**Affiliations:** ^1^ College of Medicine, Southwest Jiaotong University, Chengdu, Sichuan, China; ^2^ Clinical Biobank Center and Laboratory Animal Center, The General Hospital of Western Theater Command, Chengdu, Sichuan, China; ^3^ Department of General Surgery, The General Hospital of Western Theater Command, Chengdu, China; ^4^ College of Life Science and Engineering, Southwest Jiaotong University, Chengdu, Sichuan, China

**Keywords:** cancer, cardiovascular diseases, fibrosis, long non-coding RNA CHRF, molecular mechanism, target strategies

## Abstract

Long non-coding RNAs (LncRNA), exceeding 200 nucleotides in size, have emerged as important regulators of genes involved in multiple biological functions including cell growth, migration, invasion, drug resistance and apoptosis. They are increasingly being explored in human diseases. Notably, the recently identified LncRNA Cardiac hypertrophy-related factor (CHRF) has gained attention for its involvement in the molecular mechanisms of various diseases. CHRF was originally identified as a contributive LncRNA in cardiovascular diseases. Subsequent studies also revealed that it exerts an important role in promoting fibrosis and drug resistance. However, CHRF exhibits oncogenic functions in numerous cancers, including Non-small cell lung cancer (NSCLC), Colorectal cancer (CRC), Ovarian cancer (OC), Gastric cancer (GC), indicating its crucial roles in cancer progression. CHRF exhibits tremendous potential as both therapeutic target and diagnostic biomarker, particularly in cardiomyopathy, fibrosis, and cancer. To enhance our comprehensive understanding, this review synthesizes the pathophysiological mechanisms associated with CHRF and discusses its biological significance and clinical implication. Additionally, This review provides a comprehensive discussion on therapeutic strategies based on Non-coding RNA targets and discuss the potential of targeting CHRF, which is expected to offer readers a research approach for identifying the correct target strategies.

## 1 Introduction

Long non-coding RNAs (LncRNAs) are RNA molecules longer than 200 base pairs that are not translated into proteins (Dahariya et al., 2019; Quinn and Chang, 2016). Initially, as a by-products of RNA polymerase II transcription, LncRNAs have no biological function (Schier and Taatjes, 2020; Mattick et al., 2023). However, an increasing body of evidence have demonstrated that LncRNAs regulate various cellular processes (Ransohoff et al., 2018), including chromosomal and genomic modification, transcriptional activation and interference, and nuclear transport (Dykes and Emanueli, 2017; Ferrer and Dimitrova, 2024). This has spurred further research into the role of LncRNA in human biology (Yan and Bu, 2021; Chi et al., 2019). LncRNA can be classified by their length, function, location, and targeting mechanisms, though a unified classification system has yet to be established (Matsui and Corey, 2017). According to their position relative to protein-coding genes, LncRNAs are categorized as sense, antisense, bidirectional, intronic, intergenic, or enhancer-associated (Sanchez Calle et al., 2018). Mechanistically, LncRNA are typically divided into four categories: bait, scaffold, signal, and guide (Schmitz et al., 2016). Recent advancements have highlighted that certain LncRNAs play a crucial role in encoding small peptides and modulating general biological processes in a tissue-specific manner. These findings further emphasize the intricate nature and significant biological relevance of LncRNAs (Nelson et al., 2016; Choi et al., 2019; Chen et al., 2021).

Cardiac hypertrophy-related factor (CHRF) is a recently identified LncRNA that functions as a central regulatory role in numerous cancers and diseases. It is a LncRNA with a length of 1,843 nucleotides that lacks protein-coding potential. It is genomically located on chromosome 18. While the full-length sequence of CHRF exhibits poor conservation across species, its binding site for miRNA demonstrates cross-species conservation (Wang et al., 2014). As an oncogene, CHRF is upregulated in a variety of pathological conditions, including Myocardial hypertrophy (Wang et al., 2014; Wo et al., 2018), Myocardial ischemia-reperfusion injury (Mo et al., 2021), Ischemic stroke (Gai et al., 2019), Acute lung injury (Luo et al., 2021), Silicosis (Wu et al., 2016), Idiopathic pulmonary fibrosis (Li et al., 2021), Prostate cancer (Liu S. et al., 2019), Non-small cell lung cancer (Zhang et al., 2022), Colorectal cancer (Tao et al., 2017), Ovarian cancer (Tan et al., 2020), Gastric cancer (Gong et al., 2020) and others. Given its critical role in these diseases, scientists are actively exploring the potential of CHRF as a biomarker for disease screening and working to develop technologies and drugs that target this LncRNA. However, despite CHRF’s significant impact on various diseases, the development of therapeutics targeting CHRF has lagged behind, largely due to the incomplete understanding of its underlying molecular network.

We provide an overview of the mechanisms by which CHRF contributes to disease progression, and discuss CHRF’s crucial role in cardiovascular diseases, fibrotic conditions, and cancer. Additionally, we summarize the recent advances based on non-coding RNA (ncRNA) and explore the potential and challenges of targeting LncRNA CHRF as a future therapeutic strategy. In conclusion, CHRF has garnered significant attention as a highly promising therapeutic target for clinically relevant diseases. Elucidating its precise mechanisms and functions in these conditions is likely to offer critical insights that could inform the development of more effective, targeted treatment strategies.

## 2 Performance of LncRNA CHRF in human diseases

CHRF is closely associated with the development and progression of cardiovascular diseases, cancer and fibrotic conditions. CHRF expression is typically closely associated with disease severity or progression. As a potential therapeutic and diagnostic target, CHRF regulates multiple key processes, which have cell proliferation, migration, invasion, epithelial-mesenchymal transition (EMT), and drug resistance. The mechanisms by which CHRF mainly involve its interactions with different microRNA (miRNA) and signaling pathways. Understanding the role of CHRF can aid in the development of novel therapies targeting CHRF. Association of CHRF with diseases is summarized in Table 1, and the specific mechanisms of CHRF are detailed in the following sections.

**TABLE 1:** Performance of LncRNA CHRF in different diseases.

Disease types	Key modulators	Signal pathway	Expression	Biological significance	Target cell	Reference
Myocardial hypertrophy	Angiotensin II, Isoproterenol	CHRF/miR-489/MyD88, CHRF/miR-93/AKT3	Upregulation	Promotes proliferation	Cardiomyocytes	Wang et al. (2014), Wo et al. (2018)
Myocardial ischemia-reperfusion injury	Hypoxia, ROS	CHRF/miR-182-5p/ATG7	Upregulation	Promotes autophagy and apoptosis	H9C2	Mo et al. (2021)
Ischemic stroke	Hypoxia, ROS	CHRF/miR-126/SOX6	Upregulation	Promotes apoptosis	Neurons	Gai et al. (2019)
Acute lung injury	LPS	CHRF/miR-146/Notch1	Upregulation	Promotes apoptosis and inflammation	MPVECs	Luo et al. (2021)
Silicosis	Silicon dioxide	CHRF/miR-489/MyD88/Smad3	Upregulation	Promotes inflammation and fibrosis	Macrophages, Fibroblasts	Wu et al. (2016)
Idiopathic pulmonary fibrosis	TGF-β1	CHRF/miR-146a/L1CAM	Upregulation	Promotes fibrosis	A549	Li et al. (2021)
Prostate cancer	TGF-β1	CHRF/miR-10b/Akt/NF-κB/GSK3/EMT	Upregulation	Promote proliferation and inhibit apoptosis	PC3	Liu S. et al. (2019)
Non-small cell lung cancer	IL-1β	CHRF/miR-489//MyD88	Upregulation	Promotes proliferation, migration and invasion	H460	Zhang et al. (2022)
Colorectal cancer	—	CHRF/miR-489/Twist1/EMT	Upregulation	Promote proliferation and migration	HCT116, SW480	Tao et al. (2017)
Ovarian cancer	Cisplatin	CHRF/miR-10b/STAT3/EMT	Upregulation	Promote cisplatin resistance	ES2 Cells, OVCAR, and SKOV3 OC Cells	Tan et al. (2020)
Gastric cancer	—	CHRF/EMT	Upregulation	Promote migration, invasion, and EMT processes	GC	Gong et al. (2020)

### 2.1 LncRNA CHRF in cardiovascular diseases

Myocardial hypertrophy and myocardial ischemia-reperfusion (I/R) injury are prevalent pathological conditions in cardiovascular diseases.

Myocardial hypertrophy is a compensatory response to various cardiac stresses, but if it persists, it can lead to heart failure (Heinzel et al., 1985). Emerging evidence has demonstrated that the long non-coding RNA CHRF is implicated in the regulation of cardiac hypertrophy (Wang et al., 2014; Wo et al., 2018; Zhou et al., 2019; Shen et al., 2017). Isoproterenol (Iso), which stimulates the sympathetic nervous system, induces changes in cardiomyocyte proteins, glycogen, collagen, and lipids, leading to myocardial fibrosis and hypertrophy, exacerbating the condition. Research indicates that CHRF is upregulated in Iso-induced myocardial hypertrophy models and promotes hypertrophy through the miR-93/AKT3 axis in stimulated cardiomyocytes (Wo et al., 2018). Additionally, CHRF is upregulated in hypertrophic mouse and human heart failure samples. Recent *in vitro* studies have demonstrated that miR-489 functions as an anti-hypertrophic microRNA (Wang et al., 2014). CHRF has been shown to directly bind to miR-489, thereby downregulating its expression. MyD88, a validated target of miR-489, is known to promote hypertrophic responses. The anti-hypertrophic effect of miR-489 is mediated through the inhibition of MyD88 expression. Thus, CHRF functions as an endogenous sponge to inhibit miR-489 activity and affect MyD88 levels, contributing to myocardial hypertrophy (Wang et al., 2014; Zhou et al., 2019).

**FIGURE 1:**
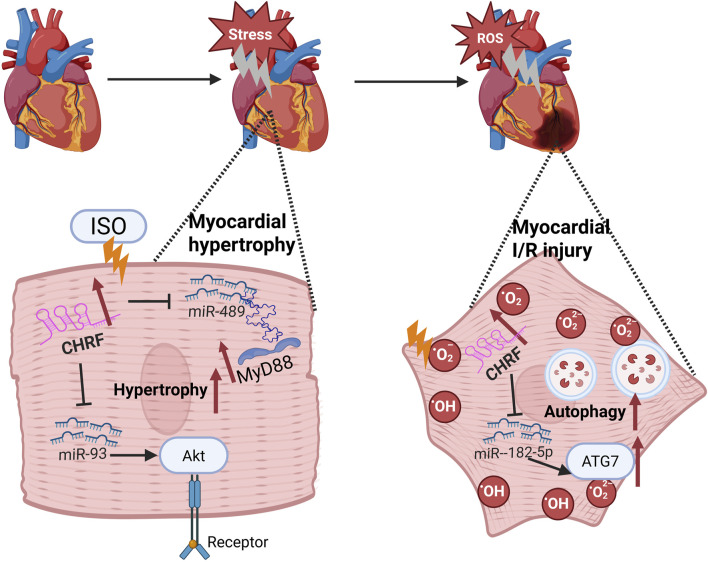
The mechanism of LncRNA CHRF in Cardiovascular diseases.

I/R injury, often caused by the restoration of blood flow after ischemia, is currently managed most effectively by early restoration of myocardial perfusion through thrombolysis (Giannakakis et al., 2017) or percutaneous coronary intervention (PCI) (Al-Lam et al., 2019), which are strategies proven to reduce infarct size and improve clinical outcomes (Li et al., 2022a). Paradoxically, the production of reactive oxygen species (ROS) can cause further damage (Hausenloy and Yellon, 2013; Davidson et al., 2019). Therefore, identifying new biomarkers for these conditions is crucial for diagnosis and treatment (Li Y. et al., 2018; Kumari et al., 2022). In the context of I/R injury, CHRF knockout significantly reduces myocardial damage, and its downregulation inhibits hypoxia/reoxygenation (H/R)-induced autophagy in cardiomyocytes. Autophagy is a vital process during myocardial ischemia-reperfusion (Popov et al., 2023; Ding et al., 2024). Bioinformatics analysis has identified miR-182-5p as a direct target of CHRF. Silencing miR-182-5p significantly enhances the effects of CHRF on cell viability, lactate dehydrogenase (LDH) levels, apoptosis, and autophagy, thereby revealing an inverse relationship between CHRF and miR-182-5p. Furthermore, autophagy-related gene 7 (ATG7), a downstream target of miR-182-5p, is positively regulated by CHRF. Overexpression of ATG7 reverses the reduction in apoptosis caused by CHRF silencing and exacerbates cardiomyocyte autophagy. This indicates that CHRF promotes myocardial ischemia-reperfusion injury by positively regulating ATG7 through the negative regulation of miR-182-5p, highlighting the therapeutic potential of targeting CHRF in treating myocardial hypertrophy and I/R injury (Solevag et al., 2020). These findings underscore the therapeutic potential of targeting CHRF in the treatment of both myocardial hypertrophy and I/R injury (Figure 1).

### 2.2 LncRNA CHRF in cancers

#### 2.2.1 Prostate cancer (PC)

Prostate cancer (PC) is the sixth most prevalent malignant tumor in men globally, with its incidence rising progressively with age (Siegel et al., 2020; Robinson et al., 2015). Elevated expression levels of CHRF have been observed in prostate cancer tissues. Notably, silencing of CHRF effectively inhibits PC cell proliferation while promoting cell apoptosis. Epithelial-mesenchymal transition (EMT) is a critical process in the development and progression of tumors, playing a significant role in tumorigenesis (Lamouille et al., 2014; Pastushenko and Blanpain, 2019). The upregulation of CHRF leads to a marked decrease in E-cadherin expression, while the levels of N-cadherin, vimentin, and ZEB1 (an EMT activator) are notably increased, thereby promoting the EMT process in prostate cancer. Furthermore, CHRF positively regulates the high expression of miR-10b in PC tissues, and inhibiting miR-10b can repress the proliferation and EMT induced by CHRF, indicating that CHRF acts in conjunction with miR-10b as an oncogene in the progression of prostate cancer. Additionally, the miR-10b/AKT/GSK3β and NF-κB pathways may be activated by CHRF, further promoting the malignant progression of PC (Liu S. et al., 2019).

#### 2.2.2 Non-small cell lung cancer (NSCLC)

Non-Small Cell Lung Cancer (NSCLC) originates from the bronchial mucosa, bronchial glands, and alveolar epithelium (Bade and Dela Cruz, 2020; Alexander et al., 2020). A recent study has underscored the crucial role of CHRF in the progression of NSCLC. Compared with adjacent normal tissues, both CHRF and interleukin-1β (IL-1β) are significantly upregulated in NSCLC tissues. IL-1β is a well-known pro-inflammatory and tumor-promoting cytokine (Weber et al., 2010), which can enhance the expression of CHRF and promote the proliferation and metastasis of NSCLC cells. Silencing CHRF can reduce the inflammatory response induced by IL-1β in NSCLC tissues. CHRF functions as a molecular sponge for microRNA-489 (miR-489), exhibiting a negative correlation with miR-489 expression. Inhabiting of miR-489 promotes the proliferation and migration of NSCLC cells, are facilitated by CHRF. Additionally, myeloid differentiation factor 88 (MyD88) is a downstream target of miR-489. MyD88 is negatively regulated by miR-489 and positively regulated by CHRF. This regulatory axis (CHRF/miR-489/MyD88) has been shown to play a significant role in the progression of NSCLC (Zhang et al., 2022).

#### 2.2.3 Colorectal cancer (CRC)

Colorectal cancer (CRC) ranks as the fourth leading cause of cancer-related mortality globally and is among the three most prevalent malignant tumors worldwide (Haggar and Boushey, 2009; Si and mon, 2016). Despite significant advancements in CRC treatment, early diagnosis remains challenging (Van Cutsem et al., 2010; Fan et al., 2021; Dekker et al., 2019). The expression of CHRF is significantly upregulated in CRC tissues, and its knockdown in CRC cells leads to reduced migration and invasion capabilities. miR-489, a target of CHRF, shows a notable decrease in expression in CRC cells, indicating a negative regulatory relationship between CHRF and miR-489 in CRC. The overexpression of miR-489 inhibits the epithelial-mesenchymal transition (EMT) process, consequently suppressing the migration and invasion of CRC cells. Emerging evidence indicates that Twist1 is a downstream target of miR-489. CHRF positively regulates Twist1 expression while negatively modulating miR-489 levels. Overexpression of Twist1 effectively counteracts the inhibitory effects of miR-489 on colorectal cancer (CRC) progression. Specifically, Twist1 promotes epithelial-mesenchymal transition (EMT), thereby facilitating CRC cell migration and invasion (Tao et al., 2017).

#### 2.2.4 Ovarian cancer (OC)

Ovarian Cancer (OC) ranks third in incidence among gynecological malignancies but has the highest mortality rate (Kujawa et al., 2015; Penny, 2020). Early diagnosis remains challenging, with the majority of patients being diagnosed at advanced stages (Penny, 2020; Orr and Edwards, 2018). Cisplatin is one of the primary chemotherapeutic agents used for the treatment of OC (Samuel et al., 2016). Studies have shown that the expression of CHRF is significantly elevated in patients with cisplatin-resistant OC, particularly in the liver, which is the first organ for OC metastasis, indicating that CHRF may play a role in cisplatin resistance in OC. It is further indicated that CHRF can directly bind to miR-10b, with a negative regulatory relationship. Silencing CHRF significantly enhances the inhibitory effect of cisplatin on OC cell proliferation, suggesting that CHRF contributes to cisplatin resistance. Moreover, this resistance is reversed when miR-10b is knocked out, reinforcing the regulatory role of the CHRF-miR-10b axis in OC. Additionally, the downregulation of CHRF leads to a significant decrease in STAT3 phosphorylation in OC cells, and this effect is similarly weakened by the upregulation of miR-10b. The CHRF-miR-10b axis exerts a significant influence on cisplatin resistance in ovarian cancer (OC) through the regulation of the epithelial-mesenchymal transition (EMT) process and the STAT3 signaling pathway (Tan et al., 2020).

#### 2.2.5 Gastric cancer (GC)

**FIGURE 2:**
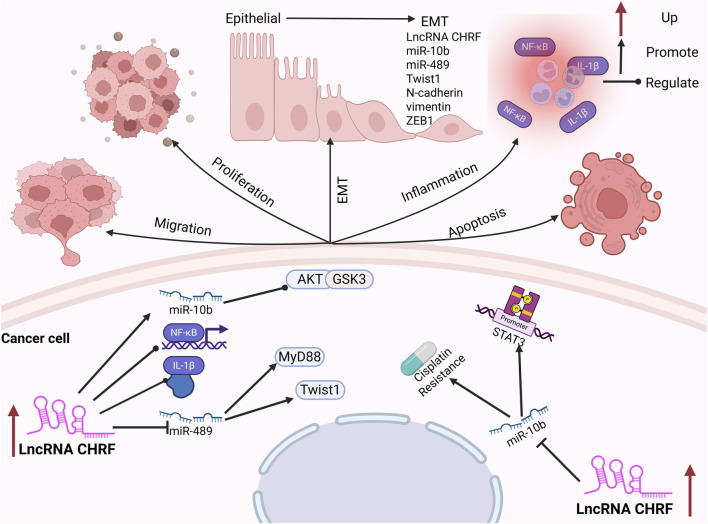
The oncogenic CHRF’s molecular mechanisms and biological roles in cancers.

Gastric Cancer (GC) originates from the gastric mucosal epithelium and is increasingly affecting younger individuals due to changes in dietary habits, increased work-related stress, and *Helicobacter pylori* infection (Thrift et al., 2023). A recent study has demonstrated that the expression of CHRF is significantly upregulated in gastric cancer (GC), particularly in cases with lymph node metastasis, where its levels are notably higher compared to non-metastatic cases. This finding indicates that CHRF plays a crucial role in promoting the progression of GC. Knocking out CHRF significantly inhibits the invasion and migration of GC cells, thereby highlighting its potential role in tumor aggressiveness. The role of CHRF in epithelial-mesenchymal transition (EMT) is of particular interest. Overexpression of CHRF suppresses the expression of the epithelial cell marker E-cadherin while promoting the expression of mesenchymal markers such as vimentin and N-cadherin. This dual effect facilitates the EMT process and contributes to the progression of GC (Gong et al., 2020) (Figure 2).

### 2.3 LncRNA CHRF in fibrotic diseases

#### 2.3.1 Silicosis

**FIGURE 3:**
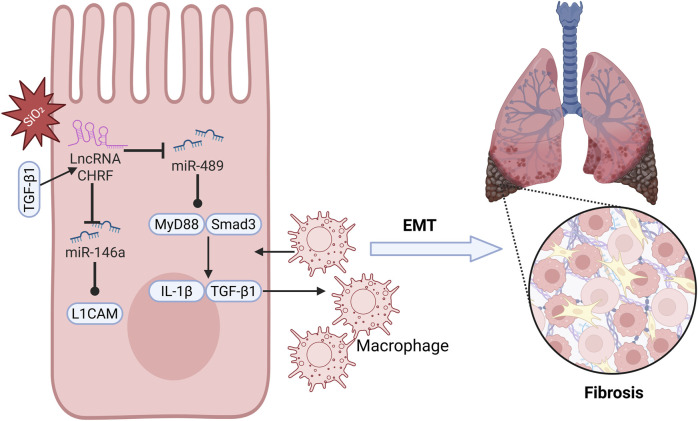
LncRNA CHRF promotes pulmonary fibrosis.

Silicosis is an incurable occupational disease caused by long-term inhalation of dust containing high concentrations of free crystalline silicon dioxide, leading to diffuse nodular pulmonary fibrosis (Chen Y. et al., 2018; Greenberg et al., 2007; Li et al., 2022b). Although silicosis is relatively common, its underlying mechanisms have not been fully explored (Tan and Chen, 2021), necessitating further research into its pathophysiological processes (Adamcakova and Mokra, 2021; Pollard, 2016). Studies indicate that miR-489 plays a role in regulating inflammation and pulmonary fibrosis associated with silicosis. Specifically, miR-489 expression is decreased in silica-induced pulmonary fibrosis, and increasing miR-489 expression significantly reduces the level of α-SMA and Vimentin proteins, suggesting that miR-489 inhibits the development of silicosis. Bioinformatics analysis identifies MyD88 and Smad3 as downstream targets of miR-489 and observes their direct interaction. Additionally, the key fibrotic factor Transforming Growth Factor-beta 1 (TGF-β1), which is produced through the activation of inflammatory macrophages and damage, is a downstream effector gene of MyD88. miR-489 may indirectly inhibit IL-1β and TGF-β1 by regulating MyD88, alleviating the inflammatory process of silicosis. Notably, CHRF exerts negative regulation on the expression of miR-489. Silencing CHRF significantly enhances the inhibitory effect of miR-489 on inflammation and fibrosis induced by silica exposure in silicosis (Figure 3). In summary, CHRF may exacerbate inflammation and fibrosis associated with silicosis through the miR-489/MyD88/Smad3 pathway (Wu et al., 2016).

#### 2.3.2 Idiopathic pulmonary fibrosis (IPF)

Idiopathic pulmonary fibrosis (IPF) is a chronic, progressive fibrosing interstitial lung disease that predominantly affects individuals in middle and older age groups (Stowasser and Hallmann, 2015; Cho and Stout-Delgado, 2020). With the increasing annual incidence of IPF and the limited survival period for patients, the situation is particularly urgent (Glass et al., 2022; Lynch et al., 2018), highlighting the pressing need to identify potential therapeutic targets for IPF (King et al., 2011). Epithelial-mesenchymal transition (EMT) is a critical process in the pathogenesis of idiopathic pulmonary fibrosis (IPF), and CHRF is a significant contributor to this process. Studies have shown that in the process of alveolar epithelial cell transformation induced by TGF-β1, CHRF is significantly upregulated in IPF cells, indicating its close relationship with the progression of IPF. Furthermore, miR-146a has been identified as a direct target of CHRF. In A549 cells treated with TGF-β1, silencing miR-146a suppresses the mRNA level of E-cadherin while enhancing the expression of Vimentin, Slug, and N-cadherin, thereby promoting EMT in lung epithelial cells and accelerating the progression of IPF. Bioinformatics analysis reveals that L1 cell adhesion molecule (L1CAM) is a downstream target of miR-146a, positively regulated by CHRF. High levels of L1CAM can reverse the inhibition of EMT observed in IPF cells when CHRF expression is reduced. In summary, as a competing endogenous RNA (ceRNA), CHRF drives the EMT process and promotes the progression of IPF by negatively regulating miR-146a and positively regulating L1CAM (Figure 3). This suggests that CHRF may become an important target for the diagnosis and treatment of IPF in the future (Li et al., 2021).

### 2.4 LncRNA CHRF in other diseases

#### 2.4.1 Ischemic stroke

Ischemic stroke, also known as ischemic apoplexy, is a condition caused by the narrowing or occlusion of cerebral arteries, leading to brain tissue necrosis and insufficient blood supply, often involving the carotid and vertebral arteries (Xu et al., 2016; Schmidt-Kastner, 2015). It progresses rapidly and is among the leading causes of disability and mortality worldwide (Green and Shuaib, 2006; Zhao et al., 2022). Research has found that after middle cerebral artery occlusion (MACO), CHRF is significantly upregulated in ischemic brain tissue. Knocking down CHRF can alleviate ischemia-reperfusion injury and improve neurological function. Bioinformatics analysis has revealed a significant correlation between CHRF and miR-126, with the upregulation of miR-126 counteracting the pro-apoptotic effects of CHRF overexpression on neurons, indicating that CHRF negatively regulates miR-126 to promote neuronal death. SOX6, acting as a downstream target of miR-126, competitively binds with CHRF, and silencing CHRF leads to reduced SOX6 levels, which in turn decreases ischemia-reperfusion injury, infarct size, and neuronal death, improving neurological function and behavioral outcomes. Therefore, the CHRF/miR-126/SOX6 axis may represent a promising therapeutic target for mitigating the progression of ischemic stroke (Gai et al., 2019).

#### 2.4.2 Acute lung injury (ALI)

Acute Lung Injury (ALI) is a severe clinical condition characterized by diffuse alveolar damage and caused by various injurious factors. These factors lead to damage of alveolar epithelial cells and capillary endothelial cells, ultimately resulting in pulmonary interstitial and alveolar edema (Parsons et al., 2005; Mokra, 2020). Sepsis is closely associated with the occurrence of ALI, triggering inflammatory and apoptotic pathways that cause alveolar epithelial injury and leakage of edema fluid (Chen Y. et al., 2018; Kumar, 2020). The long non-coding RNA (LncRNA) CHRF plays a significant role in the pathogenesis of ALI, with increased expression in septic mice, and the knockdown of CHRF can suppress inflammation and apoptosis in microvascular pulmonary endothelial cells. In septic models, silencing CHRF reduces the risk of ALI. CHRF promotes pneumonia by modulating pro-inflammatory and anti-inflammatory cytokines. Lipopolysaccharide (LPS) increases CHRF levels in ALI, suggesting that CHRF may promote ALI by inducing LPS production (Chen et al., 2010; Ehrentraut et al., 2019). Bioinformatics analysis reveals a negative correlation between CHRF and miR-146a, and the upregulation of miR-146a can counteract the adverse effects of CHRF. CHRF competitively binds with miR-146a to regulate Notch1, playing an active role in Notch1 expression. Therefore, CHRF exacerbates inflammation and apoptosis in microvascular pulmonary endothelial cells, promotes LPS production, and advances the progression of ALI by modulating the miR-146/Notch1 axis (Luo et al., 2021).

## 3 Multifunctional regulatory roles of LncRNA CHRF in diseases and therapeutics

**FIGURE 4:**
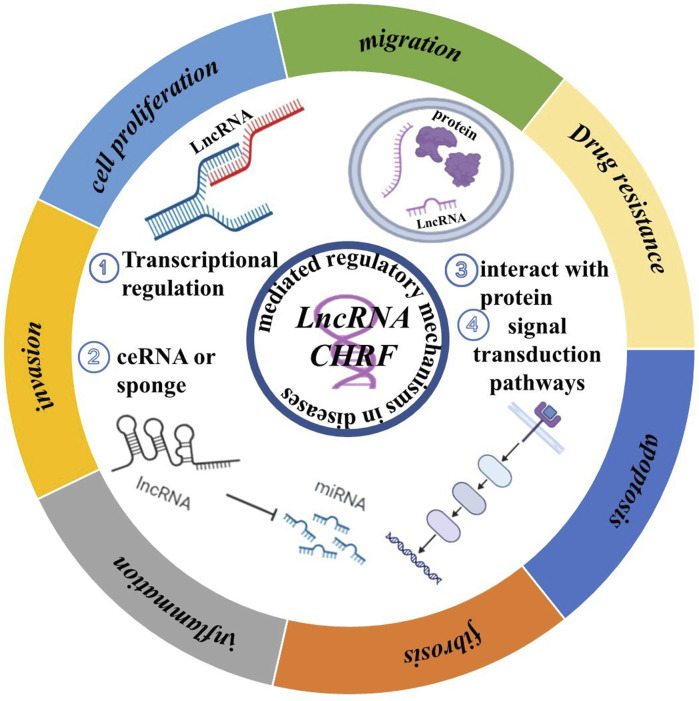
LncRNA CHRF mediated regulatory mechanisms and biological process in diseases.

CHRF is increasingly recognized for its multifaceted roles in the progression and development of diseases. As shown in Figure 4, we review the common mechanisms of CHRF across multiple diseases, aiming to highlight its significance as a drug target.

In various diseases, CHRF acts as a Competing Endogenous RNA (ceRNA) or Transcripts by binding to specific miRNAs, thereby inhibiting their function and regulating downstream gene expression. For instance, in cardiac hypertrophy, CHRF interacts with miR-93 and miR-489 to regulate AKT3 and MyD88, promoting myocardial hypertrophy. In non-small cell lung cancer and silicosis, CHRF exacerbates inflammation and fibrosis via the miR-489/MyD88/Smad3 axis. Additionally, CHRF is a crucial factor in tumors and fibrosis-related diseases by interacting with proteins, which promotes cell migration and invasion. In prostate cancer, colorectal cancer, and gastric cancer, CHRF influences Twist1 or other EMT-related factors through miR-489 or miR-10b, facilitating the EMT of cancer cells. In pulmonary fibrosis, CHRF promotes EMT and fibrosis by regulating miR-146a and L1CAM. Interestingly, CHRF contributes to cell apoptosis and tumor drug resistance. For example, in ovarian cancer, CHRF enhances cisplatin resistance through the regulation of the miR-10b and STAT3 pathways. In myocardial ischemia-reperfusion injury, CHRF modulates autophagy and apoptosis via the miR-182-5p/ATG7 pathway. Besides, CHRF exhibits significant regulatory roles in inflammatory and fibrotic diseases, particularly in acute lung injury, silicosis, and idiopathic pulmonary fibrosis. In these conditions, CHRF promotes inflammatory responses and fibrotic processes by regulating factors such as miR-489, MyD88, and Smad3.

CHRF also exerts its effects in various diseases through similar miRNA regulatory axes, including the CHRF/miR-489/MyD88 Axis, which is involved in cardiac hypertrophy, non-small cell lung cancer, and silicosis and is often associated with inflammatory responses, cell proliferation, and fibrosis. In the CHRF/miR-93/AKT3 Axis, CHRF promotes myocardial hypertrophy in cardiac hypertrophy through this regulatory pathway (Figure 4).

## 4 Targeting non-coding RNA for drug development

Non-coding RNAs, including long LncRNAs and MicroRNAs, have emerged as critical regulators in the pathogenesis and progression of various diseases. We have summarized the current popular methods for targeting Non-coding RNA (ncRNA), and how to select them, which include the following three approaches. Additionally, Based on LncRNA CHRF, we discuss the potential strategies targeted LncRNA CHRF.

### 4.1 Strategies for targeting non-coding RNA

#### 4.1.1 Small interfering RNA (siRNA)

**FIGURE 5:**
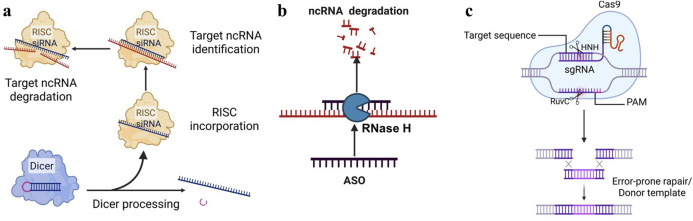
Prevalent targeted strategies of Non-coding RNA. **(a)** siRNA: siRNA specifically binds to and degrades the corresponding ncRNA, thereby preventing the continued translation of mRNA. **(b)** ASO: Forming a complementary strand with a specific ncRNA sequence, an heteroduplex is created, which is then recognized and degraded by endogenous cellular RNase H, thereby inducing gene silencing. **(c)** CRISPR/Cas9: Utilizing site-specific Cas nucleases to induce double-strand breaks (DSBs) at specific genomic loci, which are subsequently repaired via the cell’s intrinsic non-homologous end joining (NHEJ) or homologous recombination repair (HDR) pathways, ultimately enables precise genomic modifications, including gene knockout and base editing.

Small interfering RNA (siRNA) is a double-stranded RNA molecule typically comprising 20 to 25 nucleotides. It specifically targets ncRNA and subsequently recruits the RNA-induced silencing complex (RISC) to mediate the degradation of the target ncRNA (Figure 5a). Studies have shown that siRNA has been used to degrade related ncRNA in various disease models (Khorkova and Wahlestedt, 2017), demonstrating significant therapeutic potential. In esophageal squamous cell carcinoma, the use of siRNA targeting LncRNA CASC9 significantly inhibited cancer cell invasion and migration (Liang et al., 2018). With the development of drug delivery systems, the first siRNA drug, Onpattro (patisiran), for the treatment of patients with hereditary transthyretin-mediated amyloidosis and another siRNA drug, Givlaari (givosiran), approved for the treatment of adults with acute hepatic porphyria, have been launched.

#### 4.1.2 Antisense oligonucleotides (ASOs)

Antisense oligonucleotides (ASOs) are single-stranded DNA or RNA molecules that are 15–22 nucleotides in length. ASOs can bind RNA target, forming RNA/DNA heteroduplexes that are recognized and degraded by endogenous cellular RNase H, thereby inducing gene silencing (Crooke, 2017; Viney et al., 2016). It has been reported that ASOs have shown good application prospects in various diseases, especially in neurodegenerative diseases. Studies have shown that ASOs targeting MALAT1 have shown good effects in the treatment of breast cancer and multiple myeloma (Rinaldi and Wood, 2017; Arun et al., 2016). Additionally, ASO technology is characterized by its high targeting specificity, allowing for the selective inhibition of CHRF expression and preventing its pathological roles in diseases. It is also noteworthy that ASOs can inhibit transcription independently of RNase H cleavage, for example, by spatially blocking ribosome binding (Crooke, 2017). Moreover, ASOs can be chemically modified to enhance their stability and cell penetration, thereby facilitating their effective delivery into diseased tissues to inhibit ncRNA (Figure 5b). Recently, ASO-based therapies have achieved significant breakthroughs in clinical applications. To date, the U.S. Food and Drug Administration (FDA) has approved three ASO-based drugs (Rinaldi and Wood, 2017).

#### 4.1.3 CRISPR/Cas9

CRISPR/Cas9 is a widely used gene-editing tool that can target and cut specific LncRNAs (Tsai and Joung, 2014; Kopp et al., 2019; Liu et al., 2016). Single-guide RNA (sgRNA) and Cas9 enzyme are key factors in the CRISPR/Cas9 system. The CRISPR/Cas system employs site-specific Cas nucleases to introduce double-strand breaks (DSBs) at specific genomic target sites. Subsequently, the cell’s intrinsic repair mechanisms, namely, non-homologous end joining (NHEJ) or homologous recombination repair (HDR), are harnessed to repair these DSBs. This process ultimately enables precise modifications to the target gene, including gene knockout and base editing (Kopp et al., 2019; Esposito et al., 2019). Studies have shown that CRISPR/Cas9 has been used to explore the function of LncRNA (Figure 5c). For example, in prostate cancer, researchers have used CRISPR/Cas9 to knock out TTTY15, which can significantly inhibit the growth of cancer cells (Xiao et al., 2019; Chen Q. et al., 2018). This indicates that by designing a single-guide RNA (sgRNA) (Slaymaker et al., 2016; Chen et al., 2017; Koonin et al., 2023) targeting ncRNA, the Cas9 protein can recognize and cut the gene, thereby permanently blocking its expression.

However, it is important to note that the choice of targeting CHRF in preclinical models should be based on its subcellular localization within cells. ASOs are primarily employed for targeting LncRNAs located in the nucleus, whereas small interfering RNA (siRNA) is mainly used for LncRNAs in the cytoplasm. In contrast, the CRISPR/Cas9 system can be applied to LncRNAs regardless of their subcellular localization, whether in the cytoplasm or the nucleus. Importantly, the precise subcellular localization of LncRNA CHRF remains unclear, highlighting the need for further investigation. Recent studies have demonstrated the successful application of the Smart Silence method, an improved approach that combines ASOs and siRNA reagents. This method includes three siRNAs and three ASOs, and has been effectively utilized in numerous *in vivo* and *in vitro* experiments involving LncRNA knockdown (Li H. et al., 2018; Li et al., 2019; Tao et al., 2018; Liu J. et al., 2019). Therefore, for LncRNA CHRF with unclear subcellular localization, a mixed method of ASOs and siRNA should be chosen for experimentation.

### 4.2 Small molecule inhibitors

Non-coding RNAs, like proteins, possess multi-dimensional structures. Small molecule inhibitors can bind to the secondary or tertiary structures of ncRNAs, thereby blocking their interactions with proteins or other molecules (Pedram Fatemi et al., 2015). Numerous studies have demonstrated that various types of RNAs possess functional sites, including Drosha and Dicer processing sites in microRNA (miRNA) precursors, internal ribosome entry sites (IRES) in viral RNA and certain human mRNAs, riboswitches in bacteria, splicing enhancers and silencers in pre-mRNA, and regulatory structures within the 5′ and 3′ untranslated regions (UTRs). Research has further shown that small molecule inhibitors, such as ellipticine, can effectively inhibit the interaction between ncRNAs and proteins (Engreitz et al., 2014). Designing small molecule inhibitors for ncRNA involves a series of intricate steps. Initially, computational methods are employed to predict the secondary and tertiary structures of the RNA, which are then experimentally validated using techniques such as chemical modifiers and high-throughput sequencing. Following this, functional RNA structures are identified, either through computational or experimental approaches.

**FIGURE 6:**
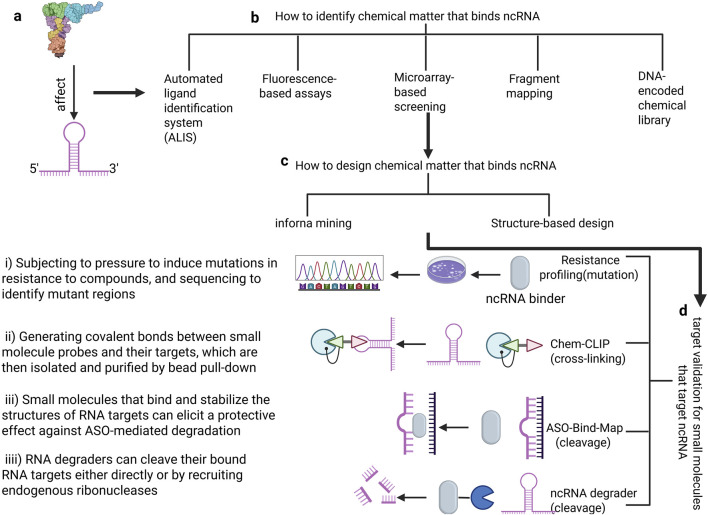
The process of designing small molecule inhibitors of Non-coding RNA. **(a)** Defining RNA structures for small-molecule targeting **(b)**. How to identify chemical matter that binds ncRNA: Including five methods (ALIS, Fluorescence-based assays, Microarray-based screening, Fragment mapping, DNA-encoded chemical library) **(c)**. How to design chemical matter that binds ncRNA: There are two prevalent strategies- infoma mining and structure-based design **(d)**. Target validation for small molecules that target ncRNA: mutation, crossing-linking, ASO-bind-Map, ncRNA degrader.

Subsequently, affinity mass spectrometry, fluorescence-based assays, and microarray screening are utilized to screen and identify small molecules capable of binding to specific RNA structures. The lead molecules obtained are then optimized through medicinal chemistry methods to enhance their pharmacological properties, including affinity, selectivity, cell permeability, and pharmacokinetics. Furthermore, the direct binding of small molecules to RNA and their biological activity must be verified, potentially involving methods such as covalent bond formation, RNA degradation, and resistance analysis. Finally, the safety and efficacy of the small molecules are assessed through preclinical and clinical studies. Throughout this process, structure-guided methods and modular assembly approaches may also be necessary to optimize the small molecules, ensuring they possess the desired pharmaceutical properties. This meticulous process ensures the development of effective small molecule inhibitors tailored for LncRNA (Childs-Disney et al., 2022) (Figure 6). Inforna is a strategy for identifying lead compounds that involves comparing the structural features present in cellular RNAs with those in experimentally determined RNA-small molecule interaction databases. The overlap identified through this comparison provides potential lead targets and lead small molecules for further investigation (Disney, 2019). Additionally, structure-based small molecule design relying on RNA or RNA-ligand complex structural models can guide the modification of interactions between RNA and small molecules (Batool et al., 2019). This offers the possibility for small molecule drugs to target ncRNAs.

### 4.3 Natural compounds

Natural compounds, such as resveratrol and curcumin, have shown potential in regulating ncRNA (Mishra et al., 2019; Xia et al., 2018; Zhang et al., 2013). For example, resveratrol alleviated Parkinson’s disease pathology by modulating MALAT1 (Xiao et al., 2019). Theoretically, these natural compounds could also regulate ncRNA’s expression or activity, reducing its promotion of cardiac hypertrophy, thus offering a natural therapeutic option. By screening and validating natural compounds that can regulate ncRNA expression or function, safe and effective drug candidates for treating diseases could be discovered.

### 4.4 The potential strategies targeted LncRNA CHRF

Increasing research has shown that LncRNA CHRF exhibits great potential in cancer, cardiac diseases, and fibrosis. While therapeutic strategies targeting it remain underdeveloped. Based on our ncRNA-targeted development strategy, we propose potential therapeutic approaches targeting LncRNA CHRF. As summarized in Figure 4, current research indicates that LncRNA CHRF influences cellular biological function in diseases, primarily through transcriptional regulation, molecular sponging, protein interactions, and signaling pathway modulation. Among these mechanisms, CHRF predominantly functions as a competitive endogenous RNA (ceRNA). In both cardiac hypertrophy and myocardial ischemia-reperfusion injury, *in vivo* and *in vitro* knockout experiments consistently demonstrate the detrimental role of CHRF in cardiac pathologies. Similarly, cancer studies reveal CHRF’s oncogenic function in patient samples and animal models, with its silencing in cellular experiments confirming tumor-promoting activities. Furthermore, CHRF silencing experiments in fibrotic and other diseases substantiate its pathogenic contributions. These collective findings provide a compelling rationale for developing CHRF-targeted therapeutics using strategies outlined in Section 4.1 (Strategies for Targeting Non-coding RNAs). Critically, CHRF’s functional impact depends on its interactions with proteins and other biomolecules. This presents opportunities to develop small-molecule inhibitors via high-throughput screening or computer-aided drug design to specifically disrupt CHRF’s interaction networks. Besides, Exploit CHRF’s structural features within disease-specific microenvironments to screen naturally derived compounds targeting this LncRNA. For instance, in cancer, CHRF accelerates tumor progression by regulating epithelial-mesenchymal transition (EMT)-related protein expression. This mechanistic insight establishes a solid foundation for designing CHRF-directed small-molecule inhibitors.

## 5 Conclusion and discussion

Research has found that CHRF (Cardiac Hypertrophy-Related Factor) is significantly upregulated in various diseases, including cardiac disorders, cancers, and fibrotic diseases. Mechanistically, CHRF acts as a competing endogenous RNA (ceRNA) in multiple pathologies, binding to specific miRNAs and inhibiting their activity. This competitive binding represents a key mechanism through which CHRF influences tumorigenesis and other pathological processes. Ultimately, CHRF impacts critical biological processes such as cell proliferation, migration, apoptosis, autophagy, drug resistance, and inflammatory responses, which are closely linked to patient prognosis.

Notably, the CHRF/miR-489/Myd88 axis is dysregulated in multiple diseases. Furthermore, epithelial-mesenchymal transition (EMT), a crucial biological process in tumor progression and fibrotic diseases, is also regulated by CHRF. However, the key modulators and biological significance of CHRF vary across different diseases (as summarized in Table 1). These findings underscore the potential of CHRF as a pivotal molecule for future research and highlight its promise as a valuable therapeutic target.

However, therapeutic strategies specifically targeting LncRNA CHRF remain largely unexplored. While the functional diversity of LncRNAs offers multiple avenues for therapeutic development, targeting strategies must be tailored to the specific mechanism of action of the individual LncRNA. Current approaches for LncRNA targeting include siRNA, ASO, CRISPR-Cas9, and the screening of small molecule inhibitors or natural compounds based on unique LncRNA structures. Studies demonstrating that CHRF knockout or silencing reverses disease progression provide a solid theoretical foundation for designing CHRF-targeted drugs. Despite this promise, the clinical translation of RNA-based therapies faces significant challenges, including insufficient specificity, inefficient delivery, and tolerability issues. Specificity problems stem primarily from off-target effects, which can arise from unintended activity in non-target cells, variations in the target sequence, or non-specific effects caused by dosing exceeding endogenous levels. Delivery challenges include the inherent instability of unmodified RNA molecules, the need to overcome the endosomal escape barrier for effective intracellular release, and the lack of carriers capable of precise targeting to specific organs or cell types. Furthermore, tolerability concerns and the combined impact of these challenges often lead to the discontinuation of RNA therapies in clinical trials due to insufficient efficacy.

Therefore, based on the current progress in LncRNA CHRF research, we propose rational hypotheses for its targeting strategies. However, despite our comprehensive review of CHRF’s mechanisms across different diseases, its specific pathophysiological roles—including its subcellular localization and upstream regulatory signals—require further elucidation.
