# Supplementary material for: LncRNA CHRF: molecular mechanisms and therapeutic potentials in cardiovascular diseases, cancers and fibrosis
Source: Front Cell Dev Biol. 2025 Jun 19;13:1573723. doi: 10.3389/fcell.2025.1573723 (PMC12222097; doi:10.3389/fcell.2025.1573723)
Supplement: Supplementary file 1 [file DataSheet1.PDF]

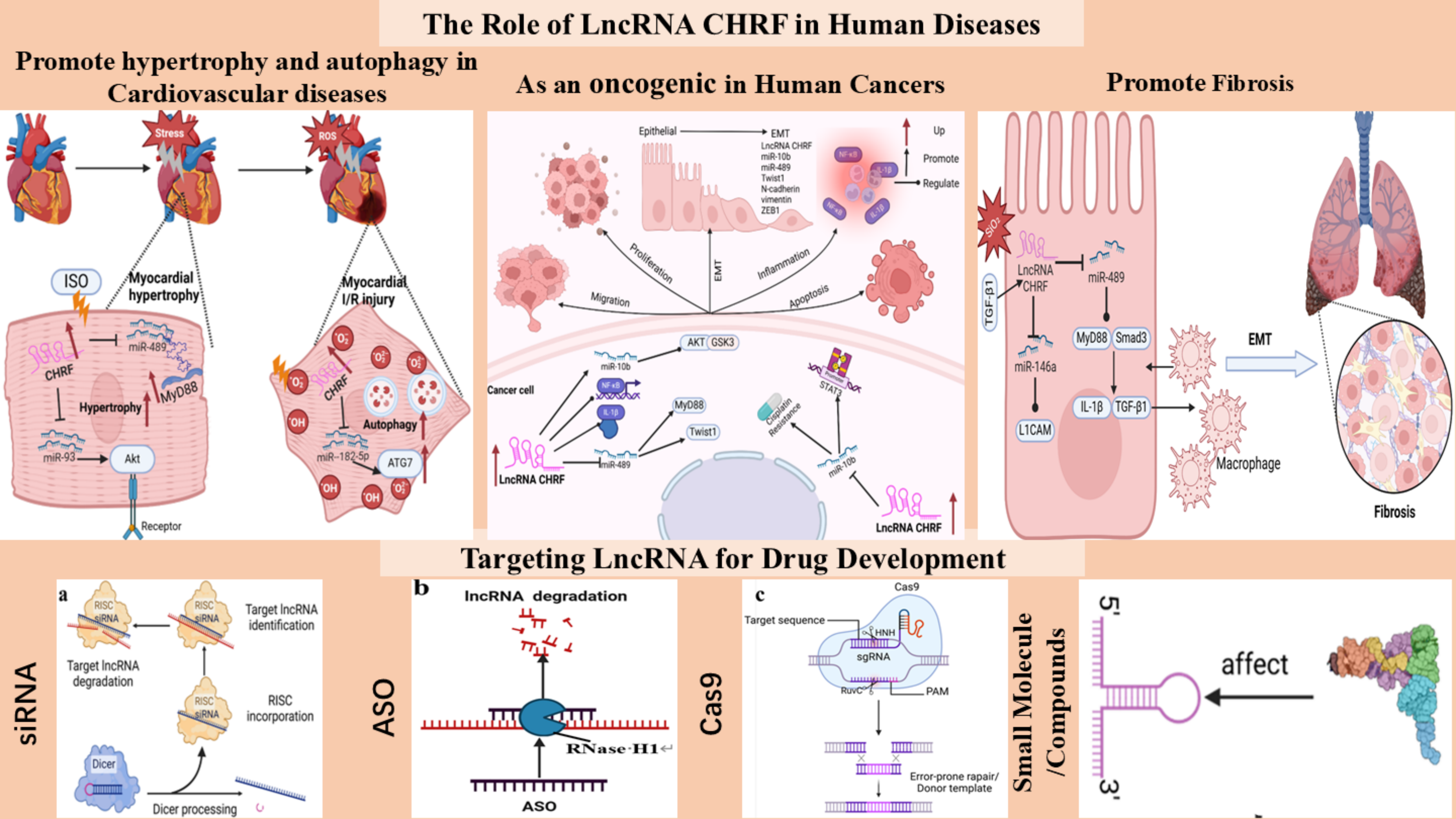
The Role of LncRNA CHRF in Human Diseases
Promote hypertrophy and autophagy in Cardiovascular diseases
Stress
ROS
ISO
Myocardial hypertrophy
Myocardial I/R injury
CHRF
miR-489
MyD88
Hypertrophy
miR-93
Akt
Receptor
miR-182-5p
Autophagy
ATG7
As an oncogenic in Human Cancers
Epithelial
EMT
LncRNA CHRF
miR-10b
miR-489
Twist1
N-cadherin
vimentin
ZEB1
Up
Promote
Regulate
NF-κB
IL-1β
Proliferation
EMT
Inflammation
Migration
Apoptosis
AKT
GSK3
Cancer cell
miR-10b
MyD88
Twist1
miR-489
LncRNA CHRF
Cisplatin Resistance
STAT3
Promoter
Promote Fibrosis
SiO2
TGF-β1
LncRNA CHRF
miR-489
miR-146a
MyD88
Smad3
L1CAM
IL-1β
TGF-β1
EMT
Macrophage
Fibrosis
Targeting LncRNA for Drug Development
siRNA
a
RISC siRNA
Target lncRNA identification
Target lncRNA degradation
RISC incorporation
Dicer
Dicer processing
ASO
b
lncRNA degradation
RNase-H1
ASO
Cas9
c
Cas9
Target sequence
HNH
sgRNA
RuvC
PAM
Error-prone rapair/ Donor template
Small Molecule /Compounds
5'
3'
affect
